# Examining the relationship between health literacy and eHealth literacy in adult populations: a systematic review and meta-analysis

**DOI:** 10.1093/heapro/daaf217

**Published:** 2025-12-16

**Authors:** Georgina Edwards, Diana Dorstyn, Melissa Oxlad

**Affiliations:** School of Psychology, The University of Adelaide, North Terrace, Adelaide, SA 5000, Australia; School of Psychology, The University of Adelaide, North Terrace, Adelaide, SA 5000, Australia; School of Psychology, The University of Adelaide, North Terrace, Adelaide, SA 5000, Australia

**Keywords:** health literacy, measure, social determinants of health, health behavior, systematic review

## Abstract

Despite their conceptual similarities and importance for effective health management, the relationship between health literacy and eHealth literacy remains poorly understood. This systematic review investigated the statistical association between health literacy and eHealth literacy in adults, along with study-level moderators and biopsychosocial correlates. CINAHL, Embase, Emcare, PubMed, ProQuest, PsycINFO, and Web of Science were searched until January 2025. Methodological reporting quality (QualSyst Checklist) was assessed and between-study heterogeneity explored using random and mixed-effects modeling. Twenty-three observational studies (*N* = 25 505 participants), all characterized by high methodological quality, were included. A weak positive relationship between overall health literacy and eHealth literacy was identified [*r* = 0.29, CI (0.21, 0.37)], with Category 2/comprehensive measures of health literacy correlating more strongly with eHealth literacy than Category 1/functional measures. Individual-level factors, including higher educational attainment, economic advantage, positive health behaviors, strong self-efficacy, and the ability to use digital resources were consistently linked to higher health literacy and eHealth literacy. The findings suggest that health literacy and eHealth literacy should continue to be researched in tandem to understand their impact on health outcomes in the digital age. Further research is also needed to understand how the surrounding environment, together with individual factors such as age and cultural background, influences the development of health literacy and eHealth literacy. Such studies are crucial for addressing disparities and enhancing access to health information and services.

Contribution to Health Promotion

- Health literacy and eHealth literacy are fundamental in healthcare.
- Researchers need to carefully choose health literacy measurement tools based on the study’s purpose and target population, to ensure reliable and relevant data.
- Environmental factors are known to influence health literacy; however, this research is less developed.

## INTRODUCTION

Definitions of health literacy typically emphasize one’s ability to find, understand, and use information and services to inform health decisions (Liu *et al*. 2020). Health literacy encompasses a combination of functional, interactive, and critical skills to access, understand, appraise, and apply health information (Nutbeam 2000, Sørensen *et al*. 2012, Liu *et al*. 2020). Arguably, a similar skillset is needed to effectively engage with online information—also referred to as eHealth literacy (Norman and Skinner 2006). Indeed, both constructs require strong reading and writing skills as well as proficiency in extracting, analyzing and using information to exert greater control over one’s own health and experiences with healthcare systems (Nutbeam 2000). However, eHealth literacy adds a crucial component: digital-specific skills, such as utilizing electronic devices and navigating websites and telehealth platforms (Nutbeam 2000). To date, however, the relationship between health literacy and eHealth literacy remains poorly understood. Individual studies have reported small or negligible correlations (Monkman *et al*. 2017, García-García *et al*. 2023), suggesting that the strength of their reciprocal influence is weak or minimal. Strong, positive correlations have also been noted—suggesting that health literacy and eHealth literacy complement one another (Neter *et al*. 2015, Singh *et al*. 2023).

These mixed findings may, in part, be attributed to variations in the measurement of each construct. Available health literacy measures typically encompass a single dimension rather than capture a constellation of skills (Karnoe and Kayser 2015). Indeed, Urstad *et al*.’s (2022) systematic review of 120 health and medical research papers revealed that most health literacy measures focused on basic reading and writing ability or disease-specific knowledge for everyday functioning (or Category 1 measures/definitions). However, definitions of health literacy should also include the ability to interpret and critically analyze health information and communicate and interact with healthcare providers (or Category 2 measures/definitions) (Urstad *et al*. 2022, p. e056294). As a result, key information relating to an individual's ability to critically analyze information and apply it to their health decisions is missing from much of the available research. eHealth literacy is, in contrast, often assessed using the eHEALS (Norman and Skinner 2006)—although the measurement of eHealth literacy itself remains an under-researched area (Délétroz *et al*. 2022).

Health literacy and eHealth literacy are both critical to effective health management, communication, and decision-making (Nutbeam and Lloyd 2021, Kim *et al*. 2023). There is some evidence that health literacy mediates the relationship between socioeconomic status and health service utilization (Stormacq *et al*. 2019). The relationship between health literacy and self-rated health or other clinical indicators (e.g. glycemic control, blood pressure) (Neter and Brainin 2019) is less clear, as is the association between health literacy and quality of life (Neter and Brainin 2019). Studies have also demonstrated a connection between eHealth literacy and improved self-management of health needs (Kim *et al*. 2023, Milanti *et al*. 2023), although much of this research has been conducted with the general population rather than individuals with chronic illnesses. Moreover, whilst eHealth literacy can enhance health outcomes, the quality of online information creates challenges (Daraz *et al*. 2019, Kim *et al*. 2023). The digital divide, which disproportionately affects low-income, elderly and rural populations due to factors such as education, cost, and infrastructure (Raihan *et al*. 2025), must also be considered to ensure equitable access to eHealth literacy skill development.

These findings highlight the importance of examining health literacy and eHealth literacy concurrently, as they are potentially interconnected and may influence one another. Understanding both provides a more complete picture of an individual's ability to navigate the healthcare landscape, including the growing trend of seeking health information online (Daraz *et al*. 2019, Wang *et al*. 2021). To date, reviews have investigated health literacy and eHealth literacy as separate concepts (Kim and Xie 2017, Neter and Brainin 2019). The current review is, to the authors’ knowledge, the first to explore the statistical association between health literacy and eHealth literacy in adult populations. A secondary aim was to identify and map the biopsychosocial correlates of health literacy and eHealth literacy and, in turn, provide a comprehensive understanding of their impact on healthcare.

## METHODS

### Search strategy and data sources

Following confirmation of search terms by a subject librarian (see Supplementary Table S1), the first author sourced relevant articles that examined the cross-sectional relationship between health literacy and eHealth literacy from the CINAHL, Embase, Emcare, PubMed, ProQuest, PsycINFO, and Web of Science databases. We conducted searches on 31 January 2025 and limited to 2006 onwards (coinciding with the year that ‘eHealth literacy’ was first coined) (Norman and Skinner 2006). In addition to searching electronic databases, we undertook Scopus citation searching and a manual screen of the reference lists of included studies to identify potentially relevant studies not indexed in core databases. A protocol for this PRISMA-guided (Page *et al*. 2021) review is available on the Open Science Framework (https://osf.io/jrwpd).

### Study eligibility

Study eligibility criteria were mapped to the following criteria, deemed relevant to association data (Moola *et al*. 2015):

1. Population: Studies that recruited an adult sample (≥18 years of age) from either the general community or a clinical cohort.
2. Outcomes: Studies that jointly evaluated health literacy and eHealth literacy using validated or purposefully designed instruments. Studies that measured one construct (health literacy or eHealth literacy, but not both), employed single-item questionnaires that do not capture the multidimensional nature of health literacy or eHealth literacy (Atanasova and Kamin 2022), or used domain-specific instruments (e.g. cancer health literacy, healthy diet literacy) were excluded.
3. Design: English-language published peer-reviewed journal articles and gray literature (i.e. dissertations) involving an observational study design (e.g. cross-sectional, case-control, longitudinal, and cohort studies) were included. Mixed or multimethod studies were also eligible if they provided correlational data. Where repeated measurements were employed (i.e. longitudinal data), only baseline data were considered, given that cross-lagged models with multiple waves of assessment can produce spurious effects (Lucas 2023). Further, only total health literacy and eHealth literacy scores were examined; inter-dimensional correlational data (e.g. HLQ item 1 correlation with eHLQ item 1; HLQ item 1 correlation with total eHEALS score) were ineligible. Finally, studies reporting non-parametric data, which can lead to inaccurate results when converted to Fisher’s *z* (Bishara and Hittner 2017), or multivariate data, which can lead to an inflated Type I error rate (Jackson *et al*. 2011), were excluded.

### Study selection

We imported identified records into Covidence software (Version 1.0, Veritas Health Innovation) for de-duplication and screening. The first two authors independently screened the records, with moderate to substantial agreement noted at each stage (title and abstract: 92% proportionate agreement, κ = 0.83; full text: 98% proportionate agreement, κ = 0.61). The few disagreements were resolved through consensus discussion.

### Data extraction

The first author independently extracted and categorized data from each included study using a purposefully designed Microsoft Excel template. The second author then verified the extraction results. We contacted the authors of two studies (Xie *et al*. 2024, Olsbø *et al*. 2025) to seek clarification or request missing or additional data, with both responding to our requests. Extracted data included: study characteristics (e.g. lead author, country of study, methodology, outcome measurements), sample parameters (e.g. demographics), biopsychosocial correlates (e.g. health conditions, personal factors, socio-environmental context), and effect size data (i.e. correlation *r*). No effect size conversions were required as all studies reported Pearson’s correlation coefficient.

### Evaluation of study reporting quality

We assessed methodological rigor and reporting bias using the 14-item QualSyst checklist for quantitative studies (Kmet *et al*. 2004). Each study was rated on the extent to which it met each criterion—fully (score of 2), partially (score of 1), not at all (score of 0), or, if not relevant to observational designs, not applicable. The summary score (total sum divided by total possible score) ranged from 0 to 1, with higher scores indicating better reporting quality. The percentage of studies meeting each criterion was also calculated. We selected a conservative cutoff score (0.75) (Kmet *et al*. 2004); however, no studies were excluded based on their quality ratings. All studies were independently rated by the first two authors, with good inter-rater agreement (85%).

### Statistical analyses

Effect size data were entered into the Comprehensive Meta-Analysis software (CMA, Version 4, Biostat Inc., Englewood, NJ, USA). Before pooling effects, individual *r*s were converted to Fisher’s *z*, weighted by each study’s inverse variance, and back-transformed into *r* (Borenstein *et al*. 2021a). Where a paper provided correlational data between multiple health literacy or eHealth literacy measures, or separate correlations for subgroups (e.g. participants from different countries in West Africa) (Ouedraogo *et al*. 2024), these data were averaged to ensure that each study contributed only a single estimate to the meta-analysis.

Confidence intervals and *P*-values were additionally calculated (DerSimonian and Laird 1986) to determine the precision and statistical significance of *r,* respectively.

A one-study-removed sensitivity analysis was performed to detect any statistical outliers. Potential for publication bias was then addressed using a funnel plot, Egger *et al*.’s (1997) regression test, and Duval and Tweedie’s (2000) trim-and-fill method.

Between-study heterogeneity was estimated using the *I*^2^ statistic, representing the proportion of variance that reflects true effects rather than sampling error, and Tau (T), the standard deviation of the true effects between studies (Borenstein 2024). Prediction intervals, which identify the dispersion of effects expected from future studies, were also calculated for any meta-analysis involving 10 or more studies (Borenstein 2023).

Finally, the moderating effects of sample (i.e. participant group: carers vs. clinical vs. community) and methodological characteristics (i.e. self-reported vs. performance-based health literacy; Category 1/functional vs. Category 2/comprehensive instruments) were examined using subgroup analyses (*Q*_B_) with a mixed-effects model (Borenstein *et al*. 2021b). We adopted Urstad *et al*.’s (2022) operationalization of health literacy to differentiate between Category 1/functional measures that focused on basic skills (i.e. reading, writing, numeracy) and Category 2/comprehensive measures, which require more advanced critical appraisal skills. To ensure data independence, the two studies that included both types of health literacy measures were excluded from this analysis (Hölgyesi *et al*. 2024, Liu *et al*. 2024). Finally, a univariate meta-regression was conducted to determine the potential role of mean sample age as a covariate in the relationship between health literacy and eHealth literacy.

Biopsychosocial correlates of health literacy and eHealth literacy were summarized in a narrative synthesis. To gain a better understanding of the importance of health literacy and eHealth literacy in biopsychosocial healthcare, correlates were mapped to the International Classification of Functioning Disability and Health (ICF) framework (World Health Organization 2001), namely body functions and structures, and environmental factors (both individual and societal). Additionally, Geyh *et al*.’s (2019) classification for personal factors of the ICF was used, which focuses on individual factors (i.e. sociodemographic factors and one’s position in the immediate social and physical context), subjective experience (i.e. feelings, thoughts, beliefs, motives), and recurrent patterns (or general patterns of experience and behavior).

## RESULTS

### Study selection

**Figure 1.**
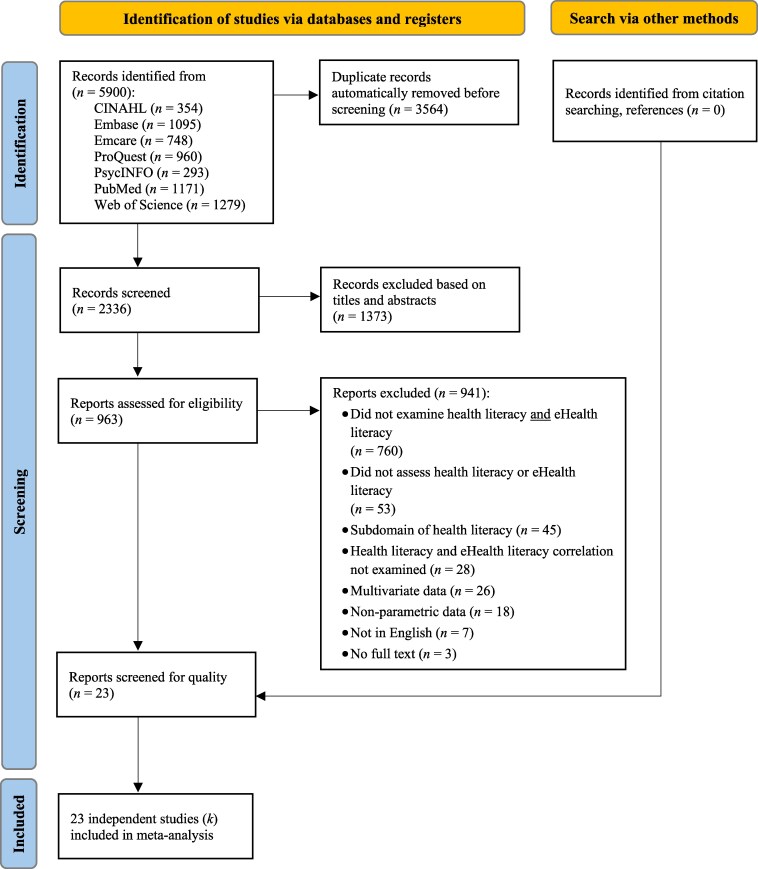
PRISMA flowchart of study selection.

A total of 5900 potentially eligible records were identified in the search process (see Fig. 1). After removing duplicates, 2336 titles and abstracts were screened for relevance, with 963 full-text reports re-screened against the eligibility criteria. No further studies were identified through citation searching or manual review of the reference lists of included studies. Two papers (Xie and Mo 2023, Xie *et al*. 2024), drawn from the same sample, were treated as one study to ensure data independence in this review (Xie *et al*. 2024). The final sample included 23 independent studies (*k*) (Monkman *et al*. 2017, Quinn 2018, Hong and Lee 2019, Do *et al*. 2020, Li *et al*. 2021, Neter *et al*. 2021, Schulz *et al*. 2021, Arriaga *et al*. 2022, Chaniaud *et al*. 2022, Efthymiou *et al*. 2022, 2025, Rastgari *et al*. 2022, Yoon *et al*. 2022, Alijanzadeh *et al*. 2023, Ashfield *et al*. 2024, Hölgyesi *et al*. 2024, Liu *et al*. 2024, Muturi and Issaka 2024, Ouedraogo *et al*. 2024, Petrič and Atanasova 2024, Sukys *et al*. 2024, Xie *et al*. 2024, Olsbø *et al*. 2025).

### Study and sample characteristics

The pooled sample comprised 25 505 participants, primarily women (63.6%, *N* = 16 214), with an average age ranging from 20 to 51 years (*M* = 34.04, *SD* = 10.75; *k* = 17) (see Table 1). Studies were published in the last seven years (from 2018 to 2025) and spanned 18 countries. There was one dissertation (Quinn 2018). Most participants were drawn from the general community (i.e. citizens, immigrants, university and school staff, and students; *k* = 16), with seven studies targeting carers (i.e. primary informal carers and health care workers; *k* = 5) or clinical cohorts (e.g. patients with Type 1 or Type 2 diabetes; *k* = 2). Most studies involved cross-sectional data (*k* = 22), with Liu *et al*. (2024) reporting baseline data from a longitudinal survey.

**Table 1.** Characteristics of included studies.

Lead author (date)	Study characteristics			Sample characteristics				Outcome measures^a^	
	Country	*N*	Recruitment source	Sub-group	Male: female ratio	Mean age (SD)	Age range	Health literacy	eHealth literacy
Alijanzadeh *et al*. (2023)	Iran	9775	Community	Citizens	0.49:1	36.4 (11.9)	NR	HELIA	eHEALS
Arriaga *et al*. (2022)	Portugal	1247	Community	Citizens	0.94:1	46 (16.7)	16–87	HLS_19_-Q12	HLS_19_-DIGI
Ashfield *et al*. (2024)	United States	219	Community	Parents of children (2–11 years)	0.18:1	NR	26–41+	Ishikawa Health Literacy Scale - modified	eHEALS
Chaniaud *et al*. (2022)	France	328	University	Students	0.2:1	21.2 (2.7)	16–33	HLS-EU-Q16	F-eHEALS
Do *et al*. (2020)	Vietnam	5209	Hospitals and Health Centers	Health care workers	0.49:1	NR	21–60	HLS-SF12	eHEALS
Efthymiou *et al*. (2025)	Greece	401	Community	Citizens	0.27:1	31 (13.5)	18–72	HLS-EU-Q16	eHEALS-GR & eHEALS-E-GR
Efthymiou *et al*. (2022)	Greece	174	Dementia Centers and Community	Carers of people with dementia	0.32:1	NR	<54–75+	HLS-EU-Q16	eHEALS
Hölgyesi *et al*. (2024)	Hungary	150	Pediatric Diabetology Centre	Parents of children with Type 1 diabetes	0.25:1	42.5 (5.8)	19–62	NVS & BHLS (4-point Likert scale)	eHEALS
Hong and Lee (2019)	Korea	138	Nursing College	Students	0.21:1	21 (NR)	NR	KHLI	eHEALS
Li *et al*. (2021)	China	1873	Universities	Students	1.07:1	19.6 (1.8)	18–25	National Health Literacy Survey Questionnaire	eHEALS
Liu *et al*. (2024)	China	947	Universities	Students	0.89:1	19.9 (1.7)	NR	s-MHLS & BHLS	eHLS-Web3.0
Monkman *et al*. (2017)	Canada	36	University	Students	0.38:1	23.6 (3.8)	18–35	NVS	eHEALS
Muturi and Issaka (2024)	United States	152	Community	African immigrants	0.63:1	34 (NR)	19–60	4 validated health literacy items	eHEALS
Neter *et al*. (2021)	Israel	683	Community	Citizens	0.61:1	49.7 (17.0)	NR	HLS-EU-16 (modified to 15 items)	eHEALS
Olsbø *et al*. (2025)	Norway	132	Hospital	Parents of children with Hirschsprung disease	0.67:1	39.8 (6.8)	NR	HLQ-p	eHEALS
Ouedraogo *et al*. (2024)	Guinea and Burkina Faso	92	Hospitals	Patients with diabetes^b^	0.64:1	NR	18–50+	BHLS	eHEALS
Quinn (2018)	Northern Ireland	60	University	Students and staff	1.5:1	27.4 (9.6)	18–59	NVS-UK	eHEALS
Petrič and Atanasova (2024)	Slovenia	1944	Community	Citizens	0.81:1	NR	18–75+	HLS_19_-Q12	eHEALS-E - modified
Rastgari *et al*. (2022)	Iran	384	Polyclinics	Patients	0.46:1	33.5 (11.8)	18–46+	NVS	eHEALS
Schulz *et al*. (2021)	Italy	362	Community	Citizens	0.39:1	35.5 (13.8)	18–66	NVS	eHEALS
Sukys *et al*. (2024)	Lithuania	332	Public schools	Physical education teachers	0.63:1	51.0 (9.9)	26–65+	HLS_19_-Q12	HLS_19_-DIGI
Xie *et al*. (2024)	China	277	Community	Older citizens	1.05:1	NR	55–70+	HLS-14	C-DHLI & eHEALS
Yoon *et al*. (2022)	Korea	590	Hospital	Citizens with and without chronic disease	0.86:1	46.5 (13.0)	20–84	NVS	K-eHEALS

^a^Measures specific to this review. ^b^Type not specified. BHLS, Brief Health Literacy Screen; C-DHLI, Chinese version of 21-item Digital Health Literacy Instrument; eHEALS, eHealth Literacy Scale; eHEALS-E-GR, extended 32-item Greek version of the eHEALS; eHEALS-E-modified, extended 20-item English version of the eHEALS; eHEALS-GR, Greek version of the eHEALS; eHLS-Web3.0, eHealth literacy scale in Web 3.0 context; F-eHEALS, French version of the eHEALS; HELIA, Health Literacy Instrument for Adults; HLQ-p, Health Literacy Questionnaire-Parent; HLS-14, 14-item Health Literacy Scale; HLS19-DIGI, Health Literacy Population Survey 2019–2021-Digital Health Literacy; HLS19-Q12, 12-item version of the Health Literacy Population Survey 2019–2021; HLS-EU-Q16, Health Literacy Survey European Questionnaire; HLS-SF12, Short-Form Health Literacy Instrument; K-eHEALS, Korean version of the eHEALS; KHLI, Korean Health Literacy Instrument; N, total sample size; NR, data not available or not reported; NVS, Newest Vital Sign; NVS-UK, United Kingdom version of the NVS; SD, standard deviation; s-MHLS, Short-form Mandarin Health Literacy Scale.

Thirteen different health literacy measures were used, with the Newest Vital Sign (NVS; *k* = 6) and the Health Literacy Survey European Questionnaire (HLS-EU-Q16; *k* = 4) being the most common (see Table 1). Conversely, eHealth literacy was frequently measured with the eHEALS (*k* = 19).

### Study reporting quality

The reporting quality of the included studies was very high, with an average score of 0.92 (*SD* = 0.08; see Supplementary Table S2 and Supplementary Figure S1). Study objectives were explicit (Criterion 1: 91% fulfilled), although study design and/or sampling strategies were not always detailed (Criteria 2–3: 52% and 61% fulfilled, respectively). Key sample characteristics (e.g. age, gender) and the measurement of health literacy and eHealth literacy were clearly defined (Criteria 4–5: > 90% fulfilled). Most studies also involved a sufficiently powered sample size (Criterion 6: 91% fulfilled) (*N* required for medium correlation > 63, α = 0.05, power = 0.80) (Cohen 1992). Finally, statistical analyses were well-detailed and transparent (Criteria 7–9: 78%–100% fulfilled), as were the findings related to health literacy and eHealth literacy (Criterion 10: 87% fulfilled).

### Meta-analyses

**Figure 2.**
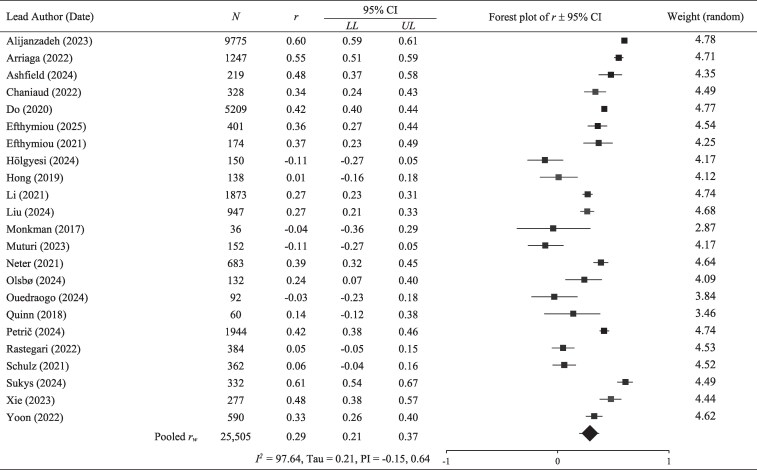
Individual study effect size *r* with forest plot for relationship between health literacy and eHealth literacy. *N* = total sample size; CI, confidence interval; LL, lower limit; UL, upper limit; *r_w_*, weighted correlation; PI, prediction interval for *r_w._*

The overall pooled effect between health literacy and eHealth literacy was significant [*r_w_* = 0.29, CI (0.21, 0.37), *P* < .001; see Fig. 2], albeit small and characterized by substantial between-study variation (*r*_range_ = −0.11–0.61; *I*^2^ = 97.64, *T* = 0.21). Indeed, the prediction interval suggested that future, similar research might report a coefficient ranging from −0.15 to 0.64.

A one-study removed analysis confirmed that no single study disproportionately impacted the results. Whilst a skewed funnel plot and significant Egger’s regression test (*P* < .001, two-tailed analysis) indicated signs of publication bias, the addition of a single imputed study (representing potential missing data) did not substantially change the magnitude of the pooled effect, suggesting the main conclusion was robust (see Supplementary Figure S2).

### Subgroup analyses

Weighted *r*s were similar regardless of the participant group sampled [*Q*_B_(2) = 3.22, *P* = .2; see Supplementary Table S3]. In comparison, performance-based tools identified weak correlations, whereas self-reported measures of health literacy produced a moderate weighted estimate [*Q*_B_(1) = 6.71, *P* = .01]. Statistically significant differences were also noted between studies that employed Category 1 and Category 2 measures [*Q*_B_(1) = 24.08, *P* < .01]. That is, health literacy measures focused on basic reading and writing skills or disease-specific knowledge and practical skills had negligible or small associations with eHealth literacy (*r*_range_ = −0.11 to 0.27). In comparison, measures that reflected the multi-dimensional nature of health literacy were positively, and often strongly, correlated with eHealth literacy (*r*_range_ = 0.01 to 0.61).

### Meta-regression

The relationship between health literacy and eHealth literacy was not significantly moderated by age for this pooled sample [*Q*_model_ = 3.82(1), *P* = .05; *R*^2^ = 0.06; *k* = 15].

### Biopsychosocial correlates of health literacy and eHealth literacy

Seventeen studies provided data relating to one or more biopsychosocial correlates of health literacy and/or eHealth literacy (see Supplementary Tables S4 and S5).

#### Body functions and structures

Five studies investigated the association between several factors related to body functions and structures and health literacy and/or eHealth literacy. Higher health literacy and eHealth literacy scores were significantly associated with a lower likelihood of suspected COVID-19 symptoms among healthcare workers (Do *et al*. 2020). In comparison, neither health literacy nor eHealth literacy varied with body mass index (Hong and Lee 2019, Sukys *et al*. 2024) or the likelihood of parents of children with Type 1 diabetes having the condition themselves (Hölgyesi *et al*. 2024). Low health literacy, but not eHealth literacy, correlated with increased vulnerability to contracting communicable diseases among African immigrants in the United States (Muturi and Issaka 2024).

#### Environmental factors

Three studies reported significant associations for health literacy and eHealth literacy and previous epidemic experience (e.g. tuberculosis, influenza, SARS) (Do *et al*. 2020), eating circumstances (e.g. eating with family, at school or delivery) (Hong and Lee 2019), and the availability of social support for physical activity (Liu *et al*. 2024). In comparison, the type of healthcare facility (i.e. frontline hospital services vs. secondary care in a health center) did not significantly impact health literacy or eHealth literacy scores (Do *et al*. 2020). Finally, physical education teachers with more work experience reported low levels of eHealth literacy compared to newly registered peers (Sukys *et al*. 2024).

#### Personal factors

##### Individual factors

The eight studies that examined this subdomain reported mixed findings. Relationships between health literacy and eHealth literacy with gender, comorbidity, college grade, employment status, marital status, socioeconomic status, residence, and household composition were largely non-significant (Hong and Lee 2019, Do *et al*. 2020, Efthymiou *et al*. 2022, Rastgari *et al*. 2022, Hölgyesi *et al*. 2024, Ouedraogo *et al*. 2024). However, economic advantage (i.e. ability to pay for medication and income) (Do *et al*. 2020, Hölgyesi *et al*. 2024, Ouedraogo *et al*. 2024) and higher educational attainment were frequently reported as important correlates of improved health literacy and eHealth literacy (Quinn 2018, Efthymiou *et al*. 2022, Rastgari *et al*. 2022, Hölgyesi *et al*. 2024, Ouedraogo *et al*. 2024). Furthermore, doctors tended to score higher on health literacy and eHealth literacy measures than other healthcare professionals (Do *et al*. 2020), as did adult children caring for older parents with dementia compared to spousal carers (Efthymiou *et al*. 2022). Ouedraogo *et al*. (2024) also reported varying correlations for both health literacy and eHealth literacy measures among diabetic patients recruited from different sub-Saharan African countries.

Notably, younger age significantly correlated with higher health literacy in some studies (Do *et al*. 2020, Efthymiou *et al*. 2022, Ouedraogo *et al*. 2024), whereas eHealth literacy demonstrated no meaningful association with age (Do *et al*. 2020, Efthymiou *et al*. 2022, Rastgari *et al*. 2022, Hölgyesi *et al*. 2024, Ouedraogo *et al*. 2024, Xie *et al*. 2024). Additionally, Efthymiou *et al*. (2022) found that support networks (i.e. having a secondary carer) played a crucial role in health literacy among primary carers, but not eHealth literacy.

##### Subjective experiences

Seven studies assessed the relationship between various subjective experiences and both health literacy and eHealth literacy (Hong and Lee 2019, Li *et al*. 2021, Neter *et al*. 2021, Rastgari *et al*. 2022, Alijanzadeh *et al*. 2023, Hölgyesi *et al*. 2024, Liu *et al*. 2024). Positive mental well-being was moderately associated with both constructs (Alijanzadeh *et al*. 2023), whereas fear of hypoglycemia in parents of children with Type 1 diabetes was not (Hölgyesi *et al*. 2024).

Studies reporting subjective measures of health yielded varied findings. Neter *et al*. (2021) identified a significant and positive relationship for both health literacy and eHealth literacy and subjective measures of health among veterans, general citizens, and immigrants, whereas a non-significant relationship was found among nursing students (Hong and Lee 2019). Self-reported COVID knowledge showed significant, albeit weak, associations with both health literacy and eHealth literacy (Li *et al*. 2021). Similarly, intention for physical activity was significantly associated with self-reported health literacy and eHealth literacy; yet, a performance-based measure of health literacy in the same study yielded the opposite result (Liu *et al*. 2024). General quality-of-life, as examined by Rastgari *et al*. (2022), was not significantly associated with health literacy or eHealth literacy scores. However, participants who perceived mental health, relationships with friends and family, and work and school as important life domains scored high on health literacy and eHealth literacy (Rastgari *et al*. 2022).

Discrepant findings were also noted for perceived risk and perceived seriousness of contracting communicable diseases (Muturi and Issaka 2024) as well as depression treatment preferences (Schulz *et al*. 2021); these variables were significantly and positively associated with health literacy but not eHealth literacy. Conversely, better self-reported health among primary care patients in Iran was significantly related (*P* < .01) to eHealth literacy, although it demonstrated a negligible relationship (*r* < 0.10) with objective, performance-based (NVS) health literacy (Rastgari *et al*. 2022). Finally, adults with higher NVS scores, but not subjective eHealth literacy (eHEALS), were able to recognize low-quality online sources of health information (Schulz *et al*. 2021).

##### Recurrent Patterns

Several noteworthy, significant relationships were identified among the 14 studies that examined the potential role of recurrent patterns—or established habits, and routines on health literacy or eHealth literacy (Quinn 2018, Hong and Lee 2019, Do *et al*. 2020, Li *et al*. 2021, Neter *et al*. 2021, Chaniaud *et al*. 2022, Efthymiou *et al*. 2022, Rastgari *et al*. 2022, Yoon *et al*. 2022, Alijanzadeh *et al*. 2023, Hölgyesi *et al*. 2024, Liu *et al*. 2024, Muturi and Issaka 2024, Sukys *et al*. 2024). Those who reported preventative health behaviors (e.g. good sleep hygiene, healthier eating, physical activity) and higher self-efficacy commonly reported higher health literacy and eHealth literacy (Hong and Lee 2019, Do *et al*. 2020, Li *et al*. 2021, Neter *et al*. 2021, Chaniaud *et al*. 2022, Rastgari *et al*. 2022, Alijanzadeh *et al*. 2023, Hölgyesi *et al*. 2024, Liu *et al*. 2024, Muturi and Issaka 2024, Sukys *et al*. 2024). However, the amount of daily sleep, diet management (e.g. regular or irregular eating pattern) (Hong and Lee 2019), and poor health habits (e.g. excessive alcohol consumption, smoking) (Hong and Lee 2019, Do *et al*. 2020, Neter *et al*. 2021, Sukys *et al*. 2024) showed mostly non-significant relationships with health literacy and eHealth literacy. Unsurprisingly, both ability and use of digital and internet-based sources to access online information (e.g. dementia resources) showed significant associations with each construct (Quinn 2018, Neter *et al*. 2021, Efthymiou *et al*. 2022, Yoon *et al*. 2022, Muturi and Issaka 2024, Sukys *et al*. 2024).

## DISCUSSION

Our review of 23 high-quality studies and a pooled sample of 25 505 adults identified an overall weak but positive relationship between health literacy and eHealth literacy. This association was moderated by the type of health literacy instrument used—although the current lack of consensus on the measurement of health literacy still needs to be overcome. Our review also identified a range of health outcomes that are strongly associated with either or both constructs.

Our findings reinforce the importance of utilizing Category 2/comprehensive measures that capture the myriad of skills required to achieve good health literacy. These measures include instruments such as the 12-item version of the Health Literacy Population Survey (HLS_19_-Q12) and the 14-item Health Literacy Scale (HLS14). However, despite recent calls for the use of Category 2/comprehensive measures in health literacy research (Urstad *et al*. 2022, Smith *et al*. 2025), Category 1/functional measures, such as the NVS, were typically used in studies in our review. The popularity of these Category 1/functional tools is likely justified by their brief administration and adaptability to research and clinical settings (Urstad *et al*. 2022). Category 1/functional tools also enable the identification of specific skill gaps for targeted interventions.

Notably, the field offers many different measurement options, with no single self-report tool being ideal for all situations. Differences in scoring methods (i.e. self-report vs performance-based tests), the varying number of questions each measure includes, and cut-off scores used to define levels of health literacy also create variations in how results are interpreted and compared across studies. The health literacy literature would benefit from investigating the timeliness and adaptability of existing Category 2/comprehensive measures at both an individual and population level (Smith *et al*. 2025). Consideration of the specific research goals (e.g. whether assessment of a specific literacy skill or a broader range of skills, contexts and environmental factors), and contextual clues known to impact on health literacy and/or eHealth literacy (e.g. the quality and accessibility of healthcare systems and information sources) will ensure that the results are interpreted appropriately and meaningfully (Jessup *et al*. 2024, Thorup *et al*. 2025). Researchers aiming for a nuanced understanding of health literacy and/or eHealth literacy should perhaps prioritize Category 2/comprehensive measures.

Our findings also highlight the important role of educational attainment and economic advantage in facilitating the development and application of health literacy and eHealth literacy. These individual factors directly influence access and use of health information and resources: higher education provides the skills needed to find, understand, and apply health information, whilst individuals with greater economic resources are generally more likely to access quality healthcare and engage effectively with online health resources (e.g. Stormacq *et al*. 2019, Aljassim and Ostini 2020, Estrela *et al*. 2023). The critical role of health-promoting behaviors and physical activity in developing good health literacy and eHealth literacy is also well established (Mantwill *et al*. 2015, Stormacq *et al*. 2019, Kim *et al*. 2023, Hua *et al*. 2025). Possessing greater health-related knowledge and skills can promote behaviors that maintain a healthy lifestyle, such as physical activity (Kinoshita *et al*. 2024). Similarly, self-efficacy, which influences how individuals approach health information and engage in self-care (Lee and Oh 2020), in addition to the ability to use digital technologies, is essential for effectively navigating the vast amount of health information that is now available online (Rastgari *et al*. 2022, Hölgyesi *et al*. 2024, Liu *et al*. 2024, Muturi and Issaka 2024). A recent review and qualitative synthesis investigating health literacy conceptualization confirmed self-efficacy as a crucial subtheme: individuals with higher self-efficacy are more likely to effectively understand and communicate their health needs and actively manage their health conditions (Liu *et al*. 2020).

Our discrepant findings include the role of self-rated health, employment status as a social determinant of health, and health-related habits (e.g. diet, sleep, smoking cessation)—all of which demonstrated weak or negligible relationships in this review. These findings contrast previous systematic and narrative reviews (Stormacq *et al*. 2019, Kim *et al*. 2023). Our results may reflect the various rating scales used by individual studies to measure self-perceived health—some of which were limited in their psychometric validity (e.g. a single self-rated health item) (Hong and Lee 2019). Our reliance on cross-sectional data also does not account for the time lag required for health literacy to translate into long-term behavior change.

Although age demonstrated a non-significant effect in our review, the narrow age range of the pooled sample (20–50) may have masked an age-related trend. There is some evidence that older individuals are at higher risk of poor health literacy and eHealth literacy, given their increased health needs and decreased reading and technology comprehension (Sørensen *et al*. 2015, Kobayashi *et al*. 2016, Hua *et al*. 2025). Other factors such as high education also provide a foundation that can empower people to make informed health decisions, regardless of age (Baccolini *et al*. 2021, Kim *et al*. 2023).

Our review identified the need to better understand the role of environmental factors in health literacy and eHealth literacy. The observational studies that we examined tended to focus on an individual’s health literacy or eHealth literacy capacities. More attention on the factors that shape health outcomes beyond individual skills and knowledge—particularly the broader physical, social, and economic environment—is needed. For example, research might examine the quality and accessibility of health information, or the role of socioeconomic and sociopolitical systems (Nutbeam and Lloyd 2021, Smith *et al*. 2025). Understanding the interplay between environmental factors and individual health literacy is crucial for creating effective interventions that address both the individual’s capacity to understand health information and the external barriers that hinder health-promoting behavior. This multi-pronged approach, combining individual support with systemic changes, is needed for people to effectively navigate increasingly complex health systems and improve outcomes.

### Limitations

Our results should be considered in the context of several methodological limitations. First, our subgroup analyses, including the distinction between Category 1/functional and Category 2/comprehensive scales, were potentially underpowered. A much higher number of studies is required to assume sufficient power when statistical heterogeneity is high (Cuijpers *et al*. 2021). Indeed, the substantial between-study heterogeneity, largely due to differing health literacy measures, introduces significant uncertainty. Second, we cannot rule out the presence of publication bias given that funnel plots are less reliable when there is substantial and real heterogeneity in the effects being studied across individual trials (Terrin *et al*. 2003, Ioannidis and Trikalinos 2007). Thus, determining whether the detected asymmetry is a result of publication bias or a real difference between smaller and larger sampled studies arising from between-group heterogeneity is unknown. Third, our meta-analysis focused on total health literacy and eHealth literacy scores, potentially oversimplifying each construct. An examination of the individual dimensions of health literacy and eHealth literacy would offer a more nuanced picture of the similarities and differences in people’s specific skills. Indeed, instruments such as the Health Literacy Questionnaire have been purposely developed to measure social dimensions and peoples’ experience of engaging with health care, including their existing abilities and resources. This strengths-based approach to health literacy measurement is argued to more accurately capture both individual and organizational capacities to address health challenges and inequalities (Osborne *et al*. 2022).

Finally, the reliance on English-language studies, limited information on cultural context among our pooled sample, and lack of representation from low and middle-income countries, restrict the generalizability of our findings to the broader adult population. Studies in our review did not include enough participants from minority groups (e.g. refugee, immigrant and indigenous populations) to perform a subgroup analysis on cultural aspects of health literacy. Minority groups (e.g. refugee, immigrant and indigenous populations) face higher risks of low health literacy and eHealth literacy due to language barriers, lack of communication and knowledge of health services, limited access to resources and opportunities, and differing views on health and illness (Stormacq *et al*. 2019, Baccolini *et al*. 2021, Estrela *et al*. 2023). Future research should consider cultural-level variability in addition to socioeconomic health disparities, given that both can impact how individuals’ access, perceive and respond to health information (Aljassim and Ostini 2020, Baccolini *et al*. 2021, Schillinger 2021).

## CONCLUSION

This review presents statistical evidence for a positive association between health literacy and eHealth literacy. The findings highlight the moderating effects of different instrument types, emphasizing the need for researchers to carefully select health literacy tools. Whilst individual factors and outcomes shared similar relationships with health literacy and eHealth literacy, the role of environmental factors requires further investigation. This research should also consider culturally and clinically diverse samples, given much research is missing from these populations. Such findings would be valuable for developing effective interventions that are tailored to individuals’ specific and changing needs to optimize their health literacy and eHealth literacy capacities.

## Supplementary Material

daaf217_Supplementary_Data

## Data Availability

The data underlying this article are available in the article and in its online supplementary material.
