# Supplementary material for: Examining the relationship between health literacy and eHealth literacy in adult populations: a systematic review and meta-analysis
Source: Health Promot Int. 2025 Dec 16;40(6):daaf217. doi: 10.1093/heapro/daaf217 (PMC12705269; doi:10.1093/heapro/daaf217)
Supplement: daaf217_Supplementary_Data [file daaf217_supplementary_data.docx]

Table S1: Database Search Terms

| **Journal** | **Health Literacy** | **eHealth Literacy** |
| --- | --- | --- |
| PubMed | “Health Literacy” [mh] OR health literac* [tiab] | eHealth literac* [tiab] OR e-health literac* [tiab] OR electronic health literac* [tiab] OR mHealth literac* [tiab] OR m-health literac* [tiab] OR mobile health literac* [tiab] OR digital health literac* [tiab] OR online health literac* [tiab] OR telehealth literac* [tiab] |
| Embase (+ Emcare) | health literacy.sh OR (health literac*).ti,ab,kf | eHealth literacy.sh OR (eHealth literac* OR e-health literac* OR electronic health literac* OR mHealth literac* OR m-health literac* OR mobile health literac* OR digital health literac* OR online health literac* OR telehealth literac*).ti,ab,kf |
| CINAHL | MH “Health Literacy+” | **TI** (“eHealth literac*” OR “e-health literac*” OR “electronic health literac*” OR “mHealth literac*” OR “m-health literac*” OR “mobile health literac*” OR “digital health literac*” OR “online health literac*” OR “telehealth literac*”) OR **AB** (“eHealth literac*” OR “e-health literac*” OR “electronic health literac*” OR “mHealth literac*” OR “m-health literac*” OR “mobile health literac*” OR “digital health literac*” OR “online health literac*” OR “telehealth literac*”) |
| PsycINFO | health literacy.sh OR (health literac*).ti,ab,id | (eHealth literac* OR e-health literac* OR electronic health literac* OR mHealth literac* OR m-health literac* OR mobile health literac* OR digital health literac* OR online health literac* OR telehealth literac*).ti,ab,id |
| Web of Science | “health literac*” | “eHealth literac*” OR “e-health literac*” OR “electronic health literac*” OR “mHealth literac*” OR “m-health literac*” OR “mobile health literac*” OR “digital health literac*” OR “online health literac*” OR “telehealth literac*” |
| ProQuest | MAINSUBJECT.EXACT(“Health literacy”) OR TI,AB,IF(“health literac*”) | TI,AB,IF(“eHealth literac*” OR “e-health literac*” OR “electronic health literac*” OR “mHealth literac*” OR “m-health literac*” OR “mobile health literac*” OR “digital health literac*” OR “online health literac*” OR “telehealth literac*”) |

Table S2: Risk of Bias in Quantitative Studies

| Lead Author (year) | 1. Question / objective sufficiently described | 2. Study design evident & appropriate | 3. Methods of subject/comparison group selection or source of information/input variables described/appropriate | 4. Subject (and comparison group) characteristics sufficiently described | 5. Random allocation to treatment group described | 6. Blinding of investigators to intervention | 7. Blinding of subjects to intervention | 8. Outcome(s) well defined and robust to measurement/ misclassification bias. Means of assessment reported. | 9. Sample size appropriate | 10. Analytic methods described/ justified/ appropriate | 11. Some estimate of variance reported | 12. Controlled for confounding | 13. Results reported in sufficient detail | 14. Conclusions supported by results | Total score (%) |
| --- | --- | --- | --- | --- | --- | --- | --- | --- | --- | --- | --- | --- | --- | --- | --- |
| Alijanzadeh (2023) | 2 | 1 | 1 | 2 | N/A | N/A | N/A | 2 | 2 | 2 | 2 | N/A | 2 | 2 | 90 |
| Arriaga (2022) | 2 | 2 | 2 | 2 | N/A | N/A | N/A | 2 | 2 | 2 | 2 | N/A | 2 | 2 | 100 |
| Ashfield (2024) | 2 | 2 | 2 | 2 | N/A | N/A | N/A | 2 | 2 | 2 | 2 | N/A | 2 | 2 | 100 |
| Chaniaud (2022) | 2 | 1 | 1 | 1 | N/A | N/A | N/A | 2 | 2 | 2 | 1 | N/A | 2 | 2 | 80 |
| Do (2020) | 2 | 2 | 1 | 2 | N/A | N/A | N/A | 2 | 2 | 2 | 2 | N/A | 2 | 2 | 95 |
| Efthymiou (2025) | 2 | 2 | 1 | 2 | N/A | N/A | N/A | 2 | 2 | 2 | 2 | N/A | 2 | 1 | 90 |
| Efthymiou (2021) | 2 | 2 | 2 | 2 | N/A | N/A | N/A | 2 | 2 | 2 | 2 | N/A | 2 | 2 | 100 |
| Hölgyesi (2024) | 2 | 2 | 2 | 2 | N/A | N/A | N/A | 2 | 2 | 2 | 2 | N/A | 2 | 2 | 100 |
| Ju-Young (2019) | 2 | 1 | 1 | 2 | N/A | N/A | N/A | 2 | 2 | 2 | 2 | N/A | 2 | 1 | 85 |
| Li (2021) | 2 | 2 | 2 | 2 | N/A | N/A | N/A | 2 | 2 | 2 | 2 | N/A | 2 | 2 | 100 |
| Liu (2024) | 2 | 2 | 2 | 2 | N/A | N/A | N/A | 2 | 2 | 2 | 2 | N/A | 2 | 2 | 100 |
| Monkman (2017) | 2 | 1 | 1 | 2 | N/A | N/A | N/A | 2 | 1 | 2 | 2 | N/A | 2 | 2 | 85 |
| Muturi (2023) | 2 | 1 | 1 | 2 | N/A | N/A | N/A | 2 | 2 | 1 | 2 | N/A | 2 | 2 | 85 |
| Neter (2021) | 2 | 1 | 2 | 2 | N/A | N/A | N/A | 2 | 2 | 2 | 2 | N/A | 2 | 2 | 95 |
| Olsbø (2024) | 2 | 2 | 2 | 2 | N/A | N/A | N/A | 2 | 2 | 2 | 2 | N/A | 2 | 2 | 100 |
| Ouedraogo (2024) | 2 | 1 | 2 | 2 | N/A | N/A | N/A | 2 | 2 | 2 | 1 | N/A | 2 | 2 | 90 |
| Quinn (2018) | 1 | 1 | 1 | 1 | N/A | N/A | N/A | 2 | 1 | 2 | 2 | N/A | 2 | 2 | 75 |
| Petrič (2024) | 2 | 1 | 2 | 2 | N/A | N/A | N/A | 2 | 2 | 2 | 1 | N/A | 2 | 2 | 90 |
| Rastegari (2022) | 2 | 1 | 2 | 2 | N/A | N/A | N/A | 2 | 2 | 2 | 2 | N/A | 2 | 2 | 95 |
| Schulz (2021) | 2 | 1 | 1 | 2 | N/A | N/A | N/A | 2 | 2 | 2 | 1 | N/A | 2 | 2 | 85 |
| Sukys (2024) | 2 | 2 | 2 | 2 | N/A | N/A | N/A | 2 | 2 | 2 | 2 | N/A | 2 | 2 | 100 |
| Xie (2023) | 2 | 2 | 2 | 2 | N/A | N/A | N/A | 2 | 2 | 2 | 2 | N/A | 2 | 1 | 95 |
| Yoon (2022) | 1 | 2 | 2 | 2 | N/A | N/A | N/A | 1 | 2 | 2 | 1 | N/A | 2 | 2 | 85 |

*Note.* Scoring criteria: 0 = not met, 1 = partially met, 2 = fully met, N/A = not applicable (not counted towards total score).

Figure S1: Assessment of Reporting Quality

Figure S2: Funnel Plot of Standard Error by Fisher’s *Z* Using Random Effects

*Note.* The black diamond represents the pooled effect with the imputed (unpublished) study

Table S3: Subgroup Analyses to Examine Sample and Methodological Characteristics

| Subgroup | Combined estimates | | | | | | |
| --- | --- | --- | --- | --- | --- | --- | --- |
|  | *k* | *N* | *r*_w_ | 95% CI | *p* | *I^2^* | *T* ^a^ |
| Participant Group |  |  |  |  |  |  |  |
| Community | 16 | 19,145 | .32 | .22 - .42 | .00 | 97.91 | .23 |
| Carers | 5 | 5,884 | .30 | .10 - .47 | .00 | 92.28 | .23 |
| Clinical | 2 | 476 | .01 | -.31 - .33 | .94 | 0.00 | .23 |
| Measurement of health literacy ^b^ | | | |  |  |  |  |
| Performance | 6 | 3,305 | .16 | .01 - .30 | .04 | 86.53 | .17 |
| Self-Report | 15 | 21,103 | .37 | .29 - .45 | .00 | 97.05 | .17 |
| Health literacy domains ^c^ | | | |  |  |  |  |
| Category 1 | 10 | 4,554 | .10 | -.00 - .21 | .06 | 88.53 | .15 |
| Category 2 | 13 | 20,951 | .42 | .35 - .39 | .00 | 96.79 | .15 |

*Note. k* = number of studies contributing to these data; *N* = pooled sample size; CI = confidence interval; *p* = p-value for *r*; *I*^2^ = proportional estimate of variance in true effects; *T =* tau, estimated standard deviation of underlying true effect across studies; ^a^ assuming a common between-study variance among subgroups of unequal groups (as per Borenstein et al., 2021). ^b^ Hölgyesi et al., 2024 & Liu et al., 2024 excluded to ensure data independence; ^c^ Category 1 = instruments describing basic reading and writing skills, disease-specific knowledge and practical skills, Category 2 = instruments describing communication and interaction skills and/or ability to interpret and critically analyze health information (Urstad et al., 2022).

Table S4: Bivariate Correlates of Health Literacy

| Author (Year) | Body Functions & Structures | Environmental Factors | | Personal Factors | | | | |
| --- | --- | --- | --- | --- | --- | --- | --- | --- |
|  |  |  |  | Individual Facts | | Subjective Experience | | Recurrent Patterns |
|  |  | Individual | Societal | Sociodemographic | Position in Immediate Social & Physical Context | Feelings | Thoughts & Beliefs / Motives | General Patterns of Experience & Behaviour |
| Alijanzadeh (2023) | - | - | - | - | - | - Mental wellbeing: *r* = .63, *p* < .001 | - | - Sleep hygiene:   *r* = .58, *p* < .001 |
| Do (2020) | - Suspected COVID-19:   *p* < .001 | - | - Epidemic experience:   *p* < .001   - Type of health care facility:   *p* = .77 | - Age: *p* = .02 - Gender: *p* < .001 - Ability to pay for medication:   *p* < .001   - Comorbidity:   *p* = .38 | - Marital status: *p* = .04 - Social status: *p* < .001 - Type of health care personnel:   *p* < .001 | - | - | - Smoking:   *p* = .05   - Drinking: *p* = .18 - Physical activity:   *p* < .001   - Dietary intake:   *p* < .001 |
| Efthymiou (2021) | - | - | - | - Age: *p* = .02 - Gender: *p* = .36 - Education: *p* = .02 - Occupation:   *p* = .07   - Socioeconomics: *p* = .12 | - Marital status: *p* = .18 - Care relationship:   *p* = .02   - Secondary carer: *p* = .02 | - | - | - Internet use:   *p* = .03   - Hours of care:   *p* = .01   - Years of care:   *p* = .32 |
| Hölgyesi (2024) | - Diabetes: NS | - | - | - Age: NS - Gender: NS - Education:   *p* < .001   - Employed: NS - Income: *p* < .05 - Comorbidity: NS | - Marital status: NS - Residence: NS ^a^; *p* < .01 ^b^ - Living in same household as T1DM child: NS | - Hypoglycaemia fear: NS | - | - Self-efficacy: *r* = -.29, *p* < .05 ^a^;   *r* = .25, *p* < .05 ^b^ |
| Ju-Young (2019) | - BMI: NS | - | - Circumstances of eating:   *p* = .04 | - Gender: NS - Grade: NS | - Residence: NS | - | - Subjective health: NS | - Smoking: NS - Drinking: NS - Physical activity: NS - Eating pattern: NS - Sleep: NS - Health behaviours: NS |
| Li (2021) | - | - | - | - | - | - | - Self-reported COVID knowledge:   *r* = .16,  *p* < .001 | - COVID-19-health related behaviours: *r* = .34, *p* < .001 - COVID-19 precautionary behaviours: *r* = .29,   *p* < .001   - Conventional health behaviours: *r* = .32,   *p* < .001 |
| Liu (2024) | - | - T2 Social support for physical activity: *r* = .37, *p* < .001 ^c^; *r* = -.07, *p* = .02 ^d^ | - | - | - | - | - T2 Intention for physical activity: *r* = .31, *p* < .001 ^c^; *r* = .03, NS ^d^ | - T2 Self-efficacy for physical activity: *r* = .29, *p* < .001 ^c^; *r* = .02, NS ^d^ - T3 Physical activity: *r* = -.08, *p* = .01 ^c^; *r* = .14, *p* < .001 ^d^ |
| Muturi (2023) | - Vulnerability to communicable diseases: *r* = .27, *p* < .01 | - | - | - | - | - | - Seriousness of communicable disease contraction: *r* = .31, *p* < .01 - Risk perception: *r* = .21, *p* < .05 | - Self-efficacy: *r* = -.22, *p* < .05 - Self-protective behaviour: NS - Response efficacy: *r* = -.29, *p* < .01 - Online health information seeking: NS - Media usage: NS - Social media usage: NS |
| Neter (2021) | - | - | - | - | - | - | - Subjective health: *r* = .33, *p* < .001 | - Smoking: NS - Physical activity: *r* = .08, *p* < .05 - Health care utilisation: *r* = -.07, *p* < .05 - Internet search outcomes: *r* = .12, *p* < .05 |
| Ouedraogo (2024) | - | - | - | - Age: *r* = -.34, *p* =.02 ^e^; *r* = -.29, *p* =.06 ^f^ - Gender: NS - Education: *r* = .92 - .94, *p* < .001 - Income: *r* = .56, *p* < .005 ^e^; *r* = .06, *p* < .005 ^f^ | - | - | - | - |
| Quinn (2018) | - | - | - | - Qualification level: *r* = .41, *p* < .01 | - | - | - | - Health question scores: *r* = .34, *p* < .01 |
| Rastegari (2022) | - | - | - | - Age: NS - Gender: NS - Education: *r* = .22, *p* < .01 | - | - | - Rate of physical health: NS - Rate of mental health: NS - Importance of work/school: *r* = .13, *p* < .01 - Importance of how you feel about yourself: NS - Importance of physical health: NS - Importance of mental health: *r* = .10, *p* < .05 - Importance of family/friends: NS - Importance of bank balance: NS | - Self-efficacy: NS |
| Schulz (2021) | - | - | - | - | - | - | - Perception of high-quality website: NS - Perception of low-quality website: *r* = -.14, *p* < .01 - Treatment preferences: *r* = .12, *p* < .01 | - |
| Sukys (2024) | - BMI: NS | - | - | - | - | - | - | - Smoking: NS - Drinking: NS - Physical activity: r = .11, p < .05 |
| Yoon (2022) | - | - | - | - | - | - | - | - Digital health technology literacy: *r* = .59, *p* < .001 - Mobile app task ability: *r* = .52, *p* < .001 |

*Note.* (-) = variable not measured; NS = non-significant association; *^a^* = data produced from the Brief Health Literacy Screener; *^b^* = data produced from the Newest Vital Sign; T2 = measured at time 2; T3 = measured at time 3; T1DM = Type 1 Diabetes Mellitus; *^c^* = data produced from self-report measure; *^d^* = data produced from performance-based measure; *^e^* = data produced from Burkina Faso sample; *^f^* = data produced from Guinea sample.

Table S5: Bivariate Correlates of eHealth Literacy

| Author (Year) | Body Functions  & Structures | Environmental Factors | | Personal Factors | | | | |
| --- | --- | --- | --- | --- | --- | --- | --- | --- |
|  |  |  |  | Individual Facts | | Subjective Experience | | Recurrent Patterns |
|  |  | Individual | Societal | Sociodemographic | Position in Immediate Social & Physical Context | Feelings | Thoughts & Beliefs / Motives | General Patterns of Experience & Behaviour |
| Alijanzadeh (2023) | - | - | - | - | - | - Mental wellbeing: *r* = .39, *p* < .001 | - | - Sleep hygiene: *r* = .36, *p* < .001 |
| Chaniaud (2022) | - | - | - | - | - | - | - | - Patient Activation: *r* = .31, *p* < .001 |
| Do (2020) | - Suspected COVID-19:   *p* < .001 | - | - Epidemic experience: *p* < .001 - Type of health care facility: *p* = .46 | - Age: *p* = .88 - Gender: *p* < .001 - Ability to pay for medication: *p* < .001 - Comorbidity: *p* = .37 | - Marital status: *p* = .29 - Social status: *p* = .27 - Type of health care personnel: *p* < .001 | - | - | - Smoking: *p* = .03 - Drinking: *p* = .53 - Physical activity:   *p* < .001  Dietary intake: *p* = .002 |
| Efthymiou (2021) | - | - | - | - Age: *p* = .11 - Gender: *p* = .85 - Education: *p* = .13 - Occupation: *p* = .03 - Socioeconomics: *p* = .07 | - Marital status: *p* = .20 - Care relationship: *p* = .03 - Caring for others: *p* = .73 - Secondary carer: *p* = .05 | - | - | - *Website use: *p* = .04 - *Social media use: *p* < .001 - *Email use: *p* < .001 - *eLearning use: *p* = .04 - *Mobile device use: *p* < .001 |
| Hölgyesi (2024) | - Diabetes: NS | - | - | - Age: NS - Gender: NS - Education: *p* = .04 - Employed: NS - Income: *p* < .05 - Comorbidity: NS | - Marital status: NS - Residence: NS - Living in same household as T1DM child: NS | - Hypoglycaemia fear: NS | - | - Self-efficacy: *r* = .35, *p* < .05 |
| Ju-Young (2019) | - BMI: NS | - | - Circumstances of eating: *p* = .01 | - Gender: *p* = .02 - Grade: NS | - Residence: *p* = .06 | - | - Subjective health: NS | - Smoking: *p* = .003 - Drinking: NS - Physical activity: *p* = .02 - Eating pattern: NS - Sleep: NS - Health behaviours: NS |
| Li (2021) | - | - | - | - | - | - | - Self-reported COVID knowledge: *r* = .22, *p* < .001 | - COVID-19-health related behaviours: *r* = .48, *p* < .001 - COVID-19 precautionary behaviours: *r* = .47, *p* < .001 - Conventional health behaviours: *r* = .36, *p* < .001 |
| Liu (2024) | - | - T2 Social support for physical activity: *r* = .53, *p* < .001 | - | - | - | - | - T2 Intention for physical activity: *r* = .51, *p* < .001 | - T2 Self-efficacy for physical activity: *r* = .57, *p* < .001 - T3 Physical activity: *r* = .09, *p* < .01 |
| Muturi (2023) | - Vulnerability to communicable diseases: NS | - | - | - | - |  | - Seriousness of communicable disease contraction: NS - Risk perception: NS | - Self-efficacy: *r* = .40, *p* < .01 - Self-protective behaviour: *r* = .24, *p* < .01 - Response efficacy: *r* = .38, *p* < .01 - Online health information seeking: *r* = .55, *p* < .01 - Media usage: NS - Social media usage: NS |
| Neter (2021) | - | - | - | - | - | - | - Subjective health: *r* = .13, *p* < .01 | - Smoking: NS - Physical activity: NS - Health care utilisation: NS - Internet search outcomes: *r* = .40, *p* < .001 |
| Ouedraogo (2024) | - | - | - | - Age: NS - Gender: NS - Education: *r* = .90 - .92, *p* < .001 - Income: *r* = .41, *p* = .004 ^e^; *r =* .42, *p* = .42 ^f^ | - | - | - | - |
| Quinn (2018) | - | - | - | - | - | - | - | - Health question scores: NS |
| Rastegari (2022) | - | - | - | - Age: NS - Gender: NS - Education: *r* = .16, *p* < .01 | - | - | - Rate of physical health: *r* = .13, *p* < .01 - Rate of mental health: *r* = .14, *p* < .01 - Importance of work/school: *r* = .12, *p* < .05 - Importance of how you feel about yourself: NS - Importance of physical health: NS - Importance of mental health: NS - Importance of family/friends: *r* = .17, *p* < .01 - Importance of bank balance: NS | - Self-efficacy: *r* = .22, *p* < .01 |
| Schulz (2021) | - | - | - | - | - | - | - Perception of high-quality website: *r* = .14, *p <* .01 - Perception of low-quality website: NS - Treatment preferences: NS | - |
| Sukys (2024) | - | - Teaching experience: *r* = -.13*, p* < .05 | - | - | - | - | - | - Use of digital resources: *r* = .11, *p* < .05 |
| Xie (2023) | - | - | - | - Age: NS | - | - | - | - |
| Yoon (2022) | - | - | - | - | - | - | - | - Digital health technology literacy: *r* = .53, *p* < .001 - Mobile app task ability: *r* = .41, *p* < .001 |

*Note.* (-) = variable not measured; NS = non-significant association; * = use for searching information related to dementia; T2 = measured at time 2; T3 = measured at time 3; T1DM = Type 1 Diabetes Mellitus; ^e^ = data produced from Burkina Faso sample; ^f^ = data produced from Guinea sample.
